# Collagen XII Contributes to Epicardial and Connective Tissues in the Zebrafish Heart during Ontogenesis and Regeneration

**DOI:** 10.1371/journal.pone.0165497

**Published:** 2016-10-26

**Authors:** Jan Marro, Catherine Pfefferli, Anne-Sophie de Preux Charles, Thomas Bise, Anna Jaźwińska

**Affiliations:** Department of Biology, University of Fribourg, Chemin du Musée 10, 1700 Fribourg, Switzerland; Northwestern University, UNITED STATES

## Abstract

Zebrafish heart regeneration depends on cardiac cell proliferation, epicardium activation and transient reparative tissue deposition. The contribution and the regulation of specific collagen types during the regenerative process, however, remain poorly characterized. Here, we identified that the non-fibrillar type XII collagen, which serves as a matrix-bridging component, is expressed in the epicardium of the zebrafish heart, and is boosted after cryoinjury-induced ventricular damage. During heart regeneration, an intense deposition of Collagen XII covers the outer epicardial cap and the interstitial reparative tissue. Analysis of the activated epicardium and fibroblast markers revealed a heterogeneous cellular origin of Collagen XII. Interestingly, this matrix-bridging collagen co-localized with fibrillar type I collagen and several glycoproteins in the post-injury zone, suggesting its role in tissue cohesion. Using SB431542, a selective inhibitor of the TGF-β receptor, we showed that while the inhibitor treatment did not affect the expression of *collagen 12* and *collagen 1a2* in the epicardium, it completely suppressed the induction of both genes in the fibrotic tissue. This suggests that distinct mechanisms might regulate collagen expression in the outer heart layer and the inner injury zone. On the basis of this study, we postulate that the TGF-β signaling pathway induces and coordinates formation of a transient collagenous network that comprises fibril-forming Collagen I and fiber-associated Collagen XII, both of which contribute to the reparative matrix of the regenerating zebrafish heart.

## Introduction

The zebrafish heart provides a valuable vertebrate model for studying cardiac development, regeneration and disease [1–3]. In adult animals, this vital organ can completely regenerate within 1 to 3 months either after removal of up to 20% of the ventricle, after cardiomyocyte-specific genetic ablation that causes the loss of up to 60% of the myocardium, or after cryoinjury-induced cardiac infarction of 20–25% of the ventricle [4–6]. Among all the standard injury procedures, cryoinjury most resembles to myocardial infarction in the mammalian heart [7–9]. Indeed, freezing/thawing leads to cell death, which triggers inflammatory responses and fibrosis in the damaged tissue. However, unlike in the injured mammalian heart, the remaining myocardium replenishes the lost tissue, while the fibrotic tissue is progressively resolved, giving space to the new myocardium.

Our laboratory has previously shown that the fibrotic matrix is essential for supporting the structure of the injured ventricular wall, as a reduction of collagen deposition results in a deformation of the ventricular wall [10]. Studies in various model systems established that the extracellular matrix not only provides a passive scaffold but also impacts cellular dynamics by regulating the availability of growth factors and cytokines and by transmitting the communication between adjacent cell types during tissue morphogenesis [11, 12]. One of the characterized ECM components in the zebrafish heart is fibronectin, which is deposited by both epicardial and fibrotic tissue cells [10, 13]. Genetic ablation of *fibronectin 1* impairs heart regeneration, although not through the regulation of cardiomyocyte proliferation [13]. In addition, a de-adhesive matrix protein, Tenascin C is deposited at the interface between the leading edge of the regenerating myocardium and the provisional wound tissue [6, 10]. Surprisingly, the contribution of specific collagenous proteins has been poorly characterized in the zebrafish heart.

The ECM organization typically involves interactions between fibril-forming collagens and fibril-associated molecules, such as proteoglycans, glycoproteins and multi-domain proteins, called fibril-associated collagens with interrupted triple helical domains (FACIT) [14, 15]. Collagen XII (Col XII), one of the FACIT proteins, is thought to form flexible bridges between adjacent collagen fibers or between collagen fibers and glycoproteins. The phenotype of *col12a1* knockout mice is characterized by muscle weakness and heavy disorganization of the bone matrix, suggesting that Col XII improves absorption of shear stress in the tissue, and consequently protects organs from mechanical distortions [16, 17]. Accordingly, human genetic studies revealed mutations in the *COL12A1* gene in patients suffering from myopathy and joint hypermobility [18, 19]. In mammals and chick, Col XII is widely expressed in the embryonic mesenchyme of various tissues, but in the adult stage, it becomes restricted to a few places, such as dermis around hair follicles, cornea, periodontal ligaments and intramuscular connective tissue [16]. In zebrafish, expression of Col XII was identified during embryogenesis at 24 and 72 hours post fertilization in the connective tissue sheaths of various organs and in certain basement membranes [20]. In this study, we addressed the contribution and regulation of this distinct non-fibrillar Col XII in the zebrafish heart in homeostatic and regenerative conditions.

## Materials and Methods

### Animal procedures

Wild-type adult zebrafish (AB, Oregon), *Tg(wt1a(-6*.*8kb)*:*GFP)*, also named *Tg(-6*.*8kbwt1a*:*GFP)* [21], and *Tg(CD41*:*EGFP)* [22] between 12–16 months were used in this study. Cryoinjuries were performed as previously described [7, 23]. Briefly, the fish were anaesthetized in 150 mg/L Tricaine (Ethyl 3-aminobenzoate methanesulfonate salt; Sigma-Aldrich, 886-86-2) dissolved in water. When the animals stopped swimming and reacting to vibrations, they were transferred onto a moist sponge. First, a small (ca. 2 mm) incision above the heart was performed and then the silvery epithelial layer of the hypodermis was removed to give direct access to the beating ventricle. A cryoprobe, precooled in liquid nitrogen for 3–5 minutes, was then positioned on the surface of the ventricle for 20–25 seconds. After this, the cryoprobe was released by pouring 2–3 mL of system water (25°C) onto the chest, and the fish were transferred to a tank with system water. After surgery, the fish were monitored for 10 min in water, and repeatedly up to 3 hours. Swimming ability was used as criterion of well-being. The animals with signs of stress and pain, such as swirling and convolutions, were euthanized. Animals in experiments were observed daily, including weekends. To sacrifice the animals, the fish were incubated for 10 min in water containing 300 mg/L Tricaine. We used loss of gill movement and tail-fin-pinch reflex to determine death. For the inhibition of TGF-β signaling, SB431542 (Tocris, 1614) was added to fish water at a concentration of 20 μM. Control animals were kept in water with 0.1% DMSO. The treatment was changed every 3 days. The cantonal veterinary office of Fribourg approved this experimental research on animals.

### Immunofluorescence

Hearts were collected and fixed in 2% paraformaldehyde, embedded in Tissue-Tek OCT compound (Sakura Finetek Europe B.V.) and cryosectioned to 16 μm thickness. Immunofluorescence staining was performed as previously described [24]. To avoid cross-reactivity of antibodies in the multiple immunofluorescence procedure, sequential staining was performed in this study. Antibodies against Col XIIa were incubated after the immunolabeling with the other antibodies.

The following primary antibodies were used: mouse anti-tropomyosin at 1:100 (DSHB, CH1), rabbit anti-Aldh1a2 at 1:200 (GeneTex, GTX124302), rabbit, anti-αSMA at 1:2000 (GeneTex, GTX100034), mouse anti-Vimentin 40E-C at 1:50 (DSHB, 40E-C), rabbit anti-Tenascin at 1:500 (USBioLogical, T2550-23), rabbit anti-Fibronectin at 1:200 (Sigma-Aldrich, F3648), rabbit anti-p-Smad3 at 1:400 (Abcam, ab52903), mouse anti-embCMHC at 1:50 (N2.261; developed by H.M. Blau, obtained from Developmental Studies Hybridoma Bank), rabbit anti-Col1a1 at 1:200 (GeneTex, GTX124368) and guinea pig anti-ColXIIa1 at 1:1000 (Bader et al, 2009). Secondary antibodies (Jackson Immunoresearch) were used at 1:500 and Phalloidin 390 (ATTO, AD 390–81) was used at 1:500.

### *In-situ* hybridization

*In-situ* hybridization on cryosections was performed as previously described [25]. The following forward (F) and reverse (R) primers (5’ to 3’) were used to synthesize antisense probes: *col12a1a* (ENSDART00000154728) F: cctgatgtctccctctaccg and R: acctggaccatgtcctctg; *col12a1b* (ENSDART00000025926), F: gagatttcgtattgaatatg and R: tcaaacgcatagagctgtgg; *col1a1a* (NM_199214.1), F: aatgagggctctgctggac and R: ggtctactgcaacaccatcg; *col1a2* (NM_182968.2), F: gaggatggctgcagtagacac and R: aatggaccaagtgtgctgtc; *tgf-β2* (NM_194385), F: acgccaaagaagtgcacaag and R: ctgtccgtatctgtggagc. The PCR products served as templates to synthesize digoxigenin-labeled RNA antisense probes using the DIG labeling system (Roche).

*In-situ* hybridization combined with immunofluorescence was performed in a sequential manner. After completion of *in-situ* hybridization staining, the slides were rinsed for 30 minutes in PBST (PBS, 0.3% Triton X-100) followed by the normal immunofluorescence protocol.

### Histological staining

A triple staining with Aniline blue, acid Fuchsin and Orange-G (AFOG) was performed as previously described [7]. For the single-dye staining with Aniline blue, cryosections were refixed with 10% neutral buffered formalin solution for 15 min and washed in PBST for 10 min. The slides were transferred into preheated Bouins fixative (Lot. 1536576, Reactorlab) and incubated for 2.5 hours at 56°C and another hour at room temperature. The slides were washed twice for 20 minutes in tap water and then incubated in 1% phosphomolybdic acid for 5 minutes. After rinsing with distilled water, sections were incubated for 4 minutes with aniline blue staining solution (1 g aniline blue diammonium salt, Sigma Aldrich, 415049; 200 ml acidified distilled water) and again washed with distilled water. The sections were dehydrated in 95% ethanol and twice in 100% ethanol for 2 minutes. After passing through three consecutive xylol baths, the slides were mounted with Entellan (107961, Merck Millipore).

### Image analysis and quantification

Fluorescent images were taken using the confocal microscopes Leica TCS SP5 and Leica TCS SP-II. Image analysis was performed using the Fiji ImageJ software and Adobe Photoshop. To quantify the percentage of Col XII in the post injured myocardium, the area positive for Col XII expression was divided by the whole post-injury myocardium area, but excluding the epicardium. N represents a number of hearts. In order to obtain representative data, at least 2 sections of each heart at different time points were imaged and analyzed. Statistics were calculated with the Prism GraphPad software. All results are expressed as the mean ± standard error of the mean (S.E.M.). Bright-field images were taken using a Zeiss Axioplan 2 microscope coupled to an AxioCam Color camera.

## Results

### Col XII-reactive fibers outline the epicardium and the subepicardium of the ventricle

**Fig 1:**
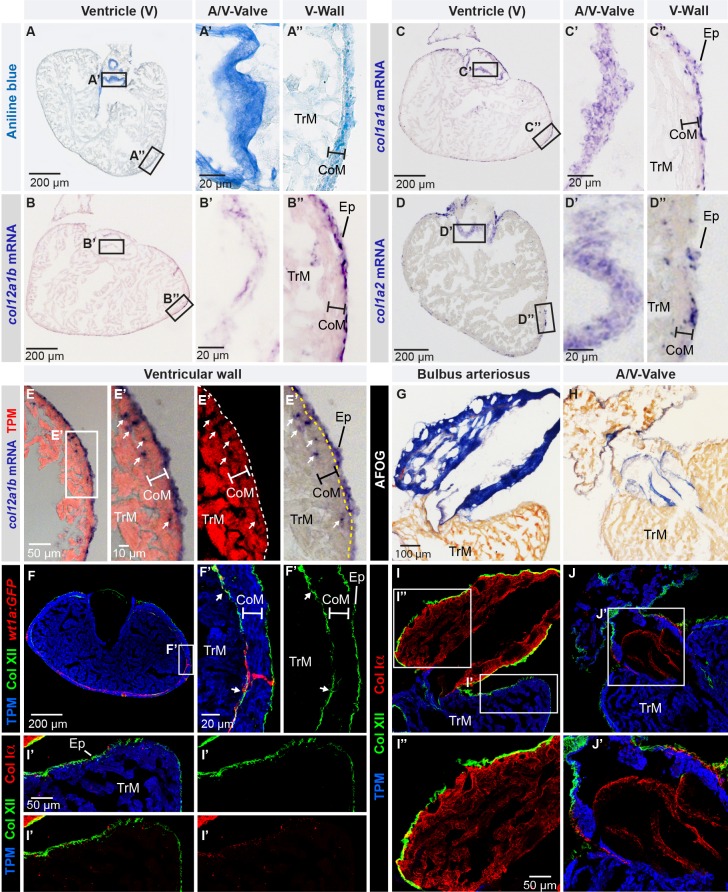
Col XII is expressed in the epicardium of the adult zebrafish heart. (A) Aniline blue staining of a ventricle transversal section visualizes collagen (blue). Framed areas encompass parts with the atrio-ventricular valve (A/V-Valve) and ventricular wall (V-Wall). The thickness of the compact myocardial layer is depicted as a bar in this and subsequent panels. Ep, epicardium; CoM, compact myocardium; TrM, trabecular myocardium. N = 5. (B-D) *In -situ* hybridization of ventricle sections detected by a color reaction (purple). Probe names are to the left. Framed areas encompass the parts that are enlarged in the panels to the right. N ≥ 4. (E) Superposition of a bright-field image with *in -situ* hybridization using *col12a1b* probe (purple) and fluorescent immunodetection of muscle protein Tropomyosin, TPM (red). *col12a1b* is expressed in the epicardium that is located externally from the myocardial border (dashed line). A few *col12a1b*-expressing cells are Tropomyosin-negative (arrows) and are interspersed within the compact myocardium (the thickness of the compact myocardium is indicated with a white bar). N = 4. (F) Immunofluorescence with anti-Tropomyosin (blue) to label cardiomyocytes and anti-Col XII (green) of transgenic fish *wt1a(-6*.*8kb)*:*GFP* (red), which labels cardiac subepicardial fibroblasts (white arrows) located mainly along the junction between the outer compact myocardium (white bar) and inner trabecular myocardium. N = 4. (G, H) Aniline blue, acid Fuchsin, Orange G (AFOG) staining detects collagen (blue) in bulbus arteriosus (G, longitudinal heart section) and the leaflets of the atrioventricular valve (H, transversal heart section). N = 6. (I, J) Triple immunofluorescence staining against Col XII (green), Col Iα (red) and Tropomyosin (blue) of the structures shown in above panels. (I’) Col XII is detected on the myocardial surface. (I”) In the bulbus arteriosus, Col Iα fibers are in the interstitium, while Col XII is restricted to its surface. (J’) The atrio-ventricular connection displays Col Iα, but little Col XII in the valve leaflets. N = 6. (A’, B’, C’, D’, E’, F’) Higher magnifications of the framed areas shown in images that are labeled with the same letter without prime symbol. The same rule applies to all subsequent figures.

In the mammalian heart, the collagenous matrix plays an important role in myocardial architecture and function [11, 12]. This subject, however, has received relatively little attention in the fish [26, 27]. In the adult zebrafish heart, histological trichrome staining (Aniline blue, acid Fuchsin and Orange G, AFOG) detected collagen by blue coloration in the non-muscular structures, namely the atrio-ventricular valve and the bulbus arteriosus of the outflow tract (S1A and S1B Fig) [28]. By contrast, little collagen staining was observed in the ventricular wall. To optimize the detection method, we used a single dye, aniline blue, without counterstaining. As expected, collagenous leaflets of the valve displayed strong blue labeling (Fig 1A and 1A’). Remarkably, the outer layer of the ventricular wall, which encompasses the epicardium and the compact myocardium, also revealed the aniline blue reactivity (Fig 1A and 1A”). This finding indicates that collagenous matrix is present along the external surface of the ventricle, which is consistent with the published electron microscopy observations [28, 29].

To determine the type of collagen that contributes to the ECM of the superficial cardiac layer, we performed *in-situ* hybridization analysis of candidate genes. In the search for an epicardial expression pattern, we identified an ortholog of the mammalian *collagen XII α1* (*col12a1)* gene, namely *col12a1b* (ZFIN database: ZDB-GENE-120215-116) (Fig 1B). The gene transcripts were detected in the outer sheaths of valve leaflets and the epicardium. The zebrafish genome contains another paralogous gene, called *col12a1a* (ZFIN database: ZDB-GENE-090728-1), which has a similar structural organization (S1D Fig). However, *in-situ* hybridization of *col12a1a* revealed weaker staining in the adult epicardium and the valve, compared to *col12a1b* (S1C Fig).

To verify whether *col12a1b*-expressing cells are non-myocytes, we performed *in-situ* hybridization in combination with immunofluorescence staining against a muscle protein, Tropomyosin. This analysis demonstrated that *col12a1b*-positive cells were devoid of Tropomyosin immunoreactivity, suggesting that they were not cardiomyocytes (Fig 1E). *col12a1b*-expressing cells formed a continuous fine layer along the external surface of the myocardium and, additionally, were interspersed across and underneath the compact myocardium (Fig 1E’). This distribution pattern and the absence of overlap with the myocyte marker suggest that *col12a1b* could be expressed not only in epicardial cells, but also in subepicardial interstitial cells.

To visualize Col XII protein distribution, we performed double immunofluorescence staining with Col XII and Tropomyosin of transgenic fish *wt1a(-6*.*8kb)*:*GFP*, which demarcates subepicardial fibroblasts [30]. Consistent with *in-situ* hybridization, Col XII antibodies covered the epicardium and at the compact/trabecular myocardial junction, which contains fibroblasts expressing *wt1a(-6*.*8kb)*:*GFP* transgenic reporter (Fig 1F and 1F’). Thus, Col XII-reactive fibers are previously uncharacterized components of the epicardial and subepicardial tissues in the zebrafish heart.

### Col XII and Col Iα are detected in the non-overlapping compartments of the intact zebrafish heart

In mammalian and avian tissues, immunogold electron microscopy and expression analyses suggested that Col XII is associated with the surface of fibril-forming collagens, such as Col Iα [31, 32]. To determine whether a similar co-distribution occurs in the zebrafish heart, we analyzed two Col Iα genes, *col1a1 and col1a2* (S1D Fig) [33]. *In-situ* hybridization of transversal sections revealed that both collagens were expressed in the valve and in the epicardium (Fig 1C and 1D). However, double immunofluorescence analysis using anti-Col XII and anti-Col Iα antibodies detected their non-overlapping expression patterns. While Col Iα immunoglobulins strongly reacted with the interstitial ECM of the non-muscular bulbus arteriosus and atrio-ventricular valve, Col XII-positive fibers were present only at the outer surface of the heart (Fig 1G–1J”). We observed only a weak dot-like pattern of the Col Iα immunoreactivity on the ventricular surface (Fig 1I’). Such a discrepancy between *in-situ* hybridization and immunoreactivity has been already observed for structural protein complexes, as discussed below. Here, we focused on the expression of matrix-bridging Col XII, which unambiguously labels extracellular fibrils within the outer layer of the zebrafish ventricle.

### Col XII is associated with the ventricle surface during development

**Fig 2:**
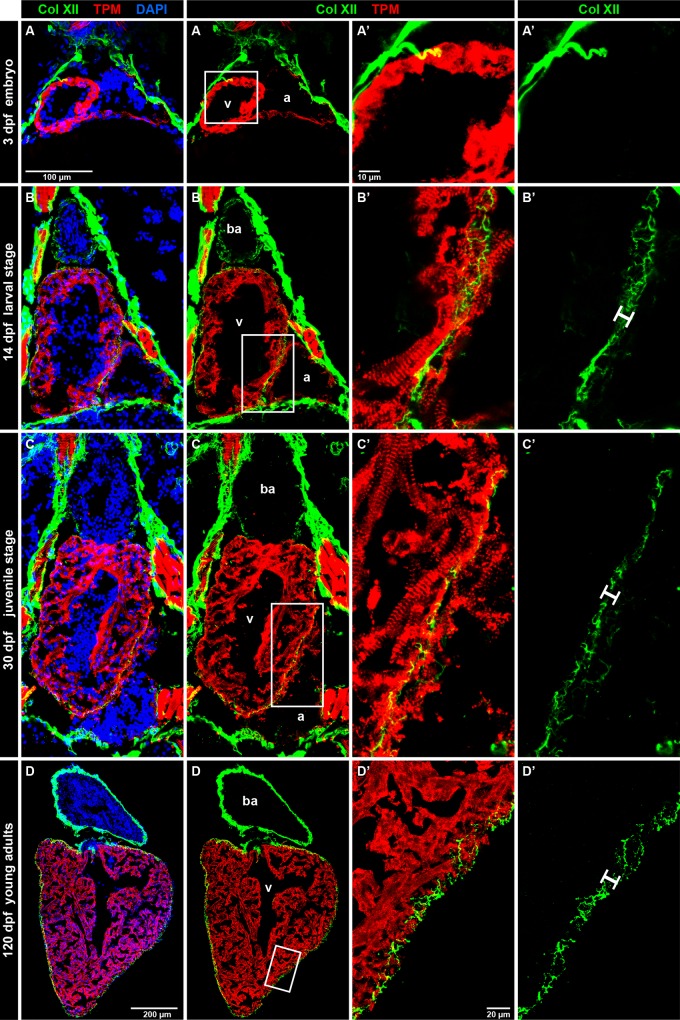
Developmental dynamics of Col XII expression in the zebrafish heart. (A-D) Longtudinal sections of the zebrafish heart after double immunostaining against Col XII (green) and Tropomyosin (red), with DAPI contrastain (blue). dpf, days post-fertilization. Three chambers of the zebrafish heart: v, ventricle; a atrium; b.a., bulbus arteriosus (non-muscular structure). N ≥ 5. (A) At 3 dpf, embryos express Col XII in the pericardium, but not in the heart. The pericardial fibers seem to invade the surface of the heart. (B) At 14 dpf, the three chambers of the larval heart are surrounded by Col XII-positive fibrils within 10 μm of outer myocardial layer (white bar). (C) At 30 dpf, the juvenile fish heart contains a thickened myocardium (red), but the size of Col XII-positive layer remains unaltered. (D) At 120 days post fertilization, young adult fish maintain Col XII-labeled fibers along the heart circumference in a pattern similar to the one seen at the larval stage.

To understand the developmental dynamics of Col XII in the zebrafish heart, we analyzed the embryonic, larval, juvenile and young adult stages, at 3, 14, 30 and 120 days post fertilization (dpf), respectively. Consistent with a previous study [20], 3-day-old embryos displayed Col XII expression in the connective tissue sheaths, but no labeling was present in the embryonic heart (Fig 2A). At this stage, nevertheless, we observed individual Col XII-positive fibrils invading the surface of the heart from the adjacent pericardial region (Fig 2A’). At 14 dpf, the outer layer of the larval heart was fully encased by Col XII-immunoreactive fibers (Fig 2B). This pattern was maintained in the juvenile and young adult zebrafish (Fig 2C and 2D). Interestingly, the Col XII-labeled structures were not only present on the surface, but also penetrated between the cardiomyocytes within the compact layer of the myocardium (Fig 2B–2D). This observation suggests that Col XII might be associated with the morphogenesis of the epicardium and subepicardium during normal growth.

### Col XII deposition is enhanced at the site of cryoinjury during zebrafish heart regeneration

The epicardium and the underlying cardiac muscle have been shown to interact during zebrafish heart regeneration [8, 34]. To determine whether Col XII is involved in this process, we used the cryoinjury model system to damage approx. 20% of the ventricle [23]. This method is based on the disruption of cell integrity by freezing/thawing of intracellular fluids that leads to cell death. We found that the original epicardial Col XII-positive fibers retained unaltered immunoreactivity along the entire ventricular circumference, including the site of injury, as shown at 0, 6 and 24 hours post-cryoinjury (hpci) (S2 Fig). This is not unexpected, as the muscle cytoskeletal proteins, namely actin filaments and associated Tropomyosin, were also detected at these time points (S2 Fig). Starting from 2 days post-cryoinjury (dpci), however, degradation of F-actin and Tropomyosin became visible, while the pre-existing Col XII fibers remained unchanged (S2 Fig). This finding indicates that certain matrix proteins, which are independent of cellular integrity, have not been destroyed by freezing/thawing, and may provide a scaffold for migration of new cells into the injury site.

**Fig 3:**
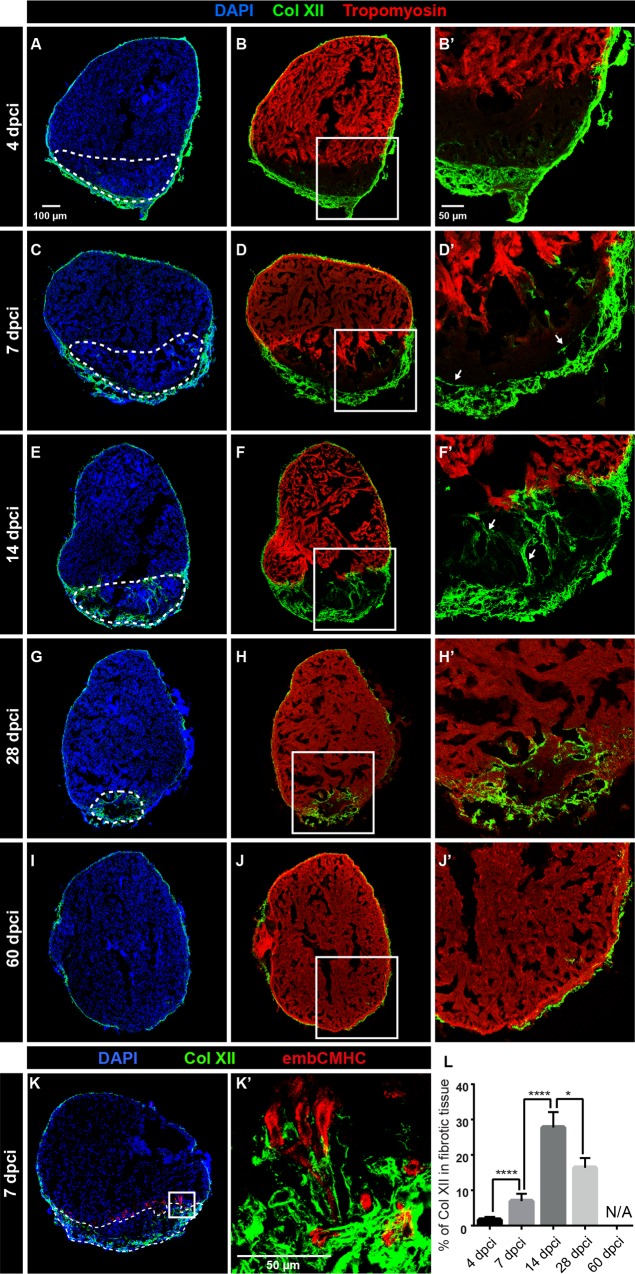
Collagen XII is transiently upregulated during heart regeneration in adult zebrafish. (A-J) Immunostaining of transversal sections of ventricles at different time points after cryoinjury. The post-infarcted tissue is defined as a Tropomyosin-negative part of the ventricle (encircled with a dashed line). (A, B) At 4 dpci, the epicardial layer with Col XII becomes markedly thicker at the site of injury. N = 8. (C, D) At 7 dpci, the border zone of the remaining myocardium contains a few Col XII-positive fibrils (arrows). N = 8. (E, F) At 14 dpci, the post-cryoinjured tissue displays accumulation of Col XII-containing matrix. N = 7. (G, H) At 28 dpci, new cardiomyocytes replace the provisional tissue, leading to the progressive decrease of the post-cryoinjured matrix. Col XII persists in the leading edge of the regenerating myocardium. N = 5. (I, J) At 60 dpci, heart regeneration has been accomplished. The expression of Col XII is reminiscent of the original pattern of uninjured hearts. N = 8. (K) Immunofluorescence staining of ventricle sections using antibodies against Col XII (green) and embryonic cardiac myosin heavy chain (embCMHC, red). At 7 dpci, undifferentiated cardiomyocytes along the regenerating leading edge are associated with the Col XII-containing ECM. N = 6. (L) The percentage of Col XII-positive area within the fibrotic tissue of the post-cryoinjured myocardium without epicardial expression. N ≥ 5; **P < 0.01, **** P < 0.0001.

The zebrafish heart repairs the cryolesion through the deposition of a transient collagenous scar as a replacement of the dead tissue [7–9]. At the same time, the remaining cardiomyocytes in the vicinity of the injury lose their differentiated character, proliferate and invade the post-infarcted area [35–37]. To assess whether Col XII contributes to the initial reparative and subsequent regenerative processes, we monitored the expression of this protein during 60 days after cryoinjury, until the zebrafish heart regeneration had been completed (Fig 3). During the reparative phase of heart regeneration, at 4 dpci, we found an increase in Col XII deposition in the epicardial region surrounding the damaged myocardium (Fig 3A–3B’). Consistently, *in-situ* hybridization demonstrated that the rapid accumulation of Col XII after injury was associated with the enhanced expression of the *col12a1a* and *col12a1b* (S3 Fig). Thus, Col XII represents a new matrix component of the thickened epicardial cap, which surrounds the damaged ventricular wall [30, 38].

At 7 dpci, Col XII-positive fibers started to accumulate in the interface between the wounded area and remaining cardiomyocytes (Fig 3C–3D’). The amount of Col XII in the center of the post-infarcted tissue increased during the more advanced regenerative phase at 14 dpci (Fig 3E–3F’). During the late regeneration stage, at 28 dpci, when a large portion of the damaged myocardium had been replaced, Col XII-fibers persisted at the junction between the new cardiomyocytes and the remnants of fibrotic tissue (Fig 3G–3H’). In the completely regenerated hearts, at 60 dpci, the pattern of Col XII immunoreactivity was reminiscent of that in uninjured hearts (Fig 3I–3J’). The dynamic deposition and resorption of Col XII indicates that this collagen type is associated with the plasticity of the transient fibrotic tissue of the regenerating heart.

The muscle-wound junction is of particular importance because this area corresponds to the regenerative leading edge [37, 39, 40]. To characterize the distribution of Col XII in relation to dedifferentiated cardiomyocytes, we used an antibody that detects an embryonic isoform of cardiac myosin heavy chain (embCMHC), which is re-expressed along the wound margin [37, 41]. Double immunostaining revealed that immature cardiac cells at the post-injury border were associated with Col XII fibers (Fig 3K and 3K’). The shape of the cells relative to the pattern of collagenous fibers suggests that the latter might contribute to the extracellular scaffold for migrating cardiomyocytes. Taken together, our data suggests that Col XII contributes to the dynamic reparative matrix that supports the regenerating myocardium.

### Heterogeneous origin of Col XII in the epicardium and fibrotic tissue

**Fig 4:**
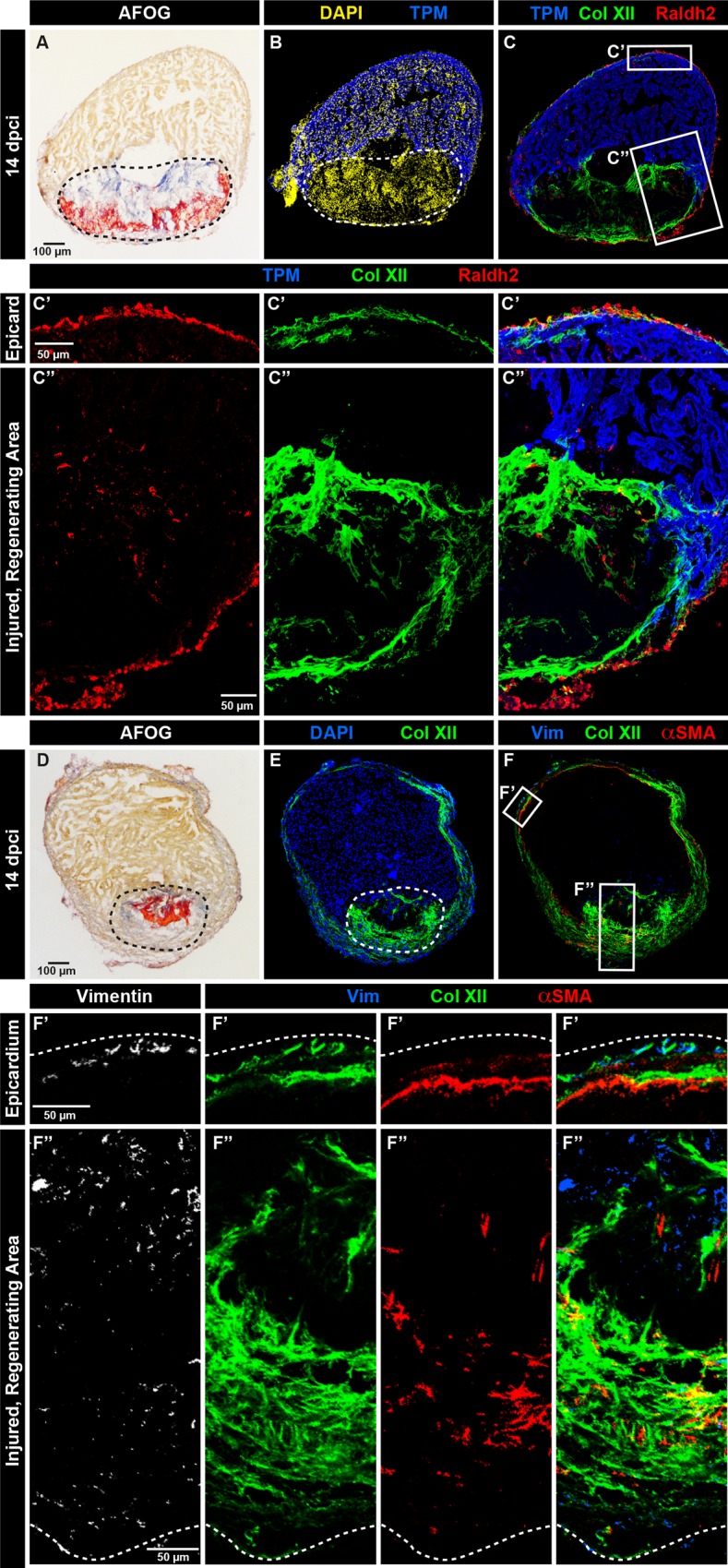
Collagen XII distribution correlates with the activated epicardium and fibroblast-like cells. (A-F) Analysis of transversal heart sections at 14 dpci. (A and D) AFOG staining of the sections used for immunostaining. (B and C) Raldh2 expression (red) demarcates the activated epicardium and endocardium. (C’) Col XII (green) and Raldh2 are colocalized in the intact epicardium (epicard). (C”) Raldh2-positive cells invade the post-cryoinjured area that is labeled by Col XII expression. Cardiac muscle is detected by Tropomyosin antibody staining (blue). N = 5. (E and F) Triple antibody staining against Col XII (green), intermediate filament Vimentin (blue) and alpha-Smooth Muscle Actin (αSMA; red). (F’ and F”) αSMA- and Vimentin-positive cells are non-overlapping cell populations in the epicardium and post-cryoinjured area. Both of them are associated with Col XII-labeled fibrils. N = 6.

Image analysis indicated that the largest area with Col XII immunoreactivity within the post-cryoinjured myocardium was observed at 14 dpci (Fig 3L). At this time point, the ECM of the damaged zone consisted of a mixture of collagen and fibrin-like material, which can be visualized using AFOG staining (Fig 4A and 4D). Thus, we selected this phase to characterize the Col XII expression in relation to participating cells and other matrix components.

To demarcate the activated epicardium, we analyzed Raldh2-expressing cells [34]. Closer examination of the ventricular surface around the uninjured and injured sites revealed a co-localization of Raldh2-positive epicardial cells and Col XII fibers (Fig 4B–4C”). Interestingly, we observed few scattered Raldh2-labeled cells in the post-cryolesion area, suggesting that the migrating epicardium-derived cells might at least partially contribute to the Col XII deposition in the fibrotic tissue (Fig 4C”).

To examine the distribution of fibroblast-like cells, we visualized Vimentin and *α*SMA, which our laboratory previously described in the context of heart regeneration (Fig 4E–4F”) [7, 10]. High magnification of the uninjured site of the ventricular surface revealed that both cytoskeletal markers are expressed in non-overlapping cell populations, namely Vimentin was detected in the outer-most epicardial layer, whereas *α*SMA labeled the junctional zone between the compact and trabecular myocardium (Fig 4F’). We found that both cell types were associated with Col XII-labeled fibers, suggesting that they might account for the deposition of Col XII along the ventricular wall. In the post-cryolesion area, Vimentin- and *α*SMA-positive cells were present in a scattered manner within the ColXII-positive region (Fig 4F”).

**Fig 5:**
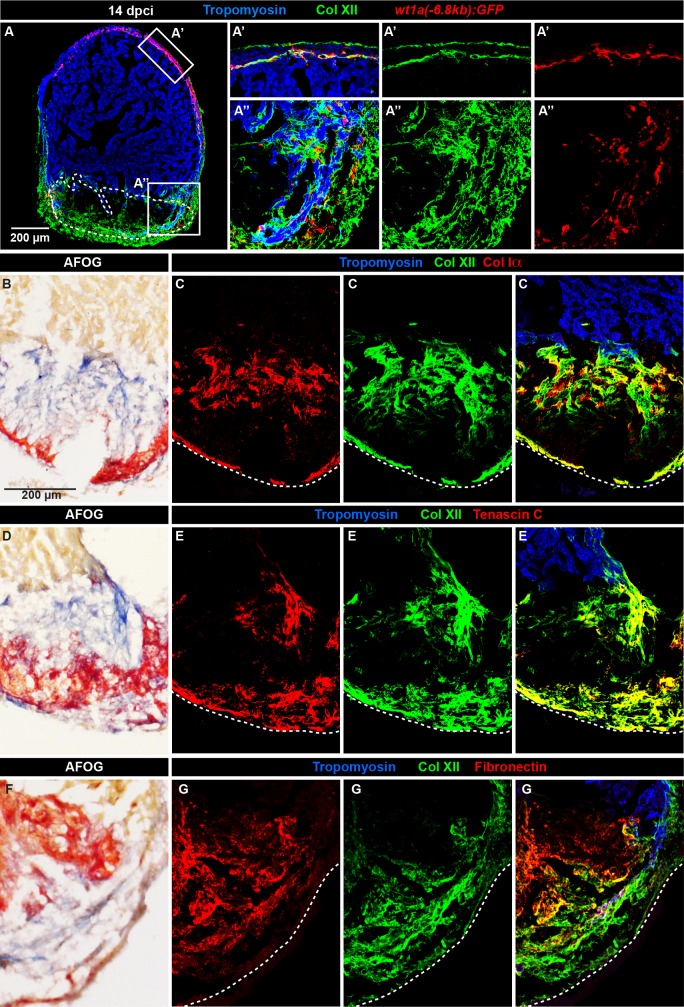
The multi-component matrix of the fibrotic post-cryoinjury tissue is rich in Collagen XII. (A) Heart section of transgenic fish *wt1a(-6*.*8kb)*:*GFP* (red) at 14 dpci. (A’) Subepicardial fibroblasts (red) co-localize with Col XII deposition (green). (A”) The injury zone contains *wt1a(-6*.*8kb)*:*GFP*-positive cardiac fibroblasts that are associated with Col XII-positive matrix. N = 5. (B-G) Higher magnifications of fibrotic wound tissue of sections at 14 dpci that were triple immunostained and then used for histological coloration with AFOG. The intact myocardium is detected by Tropomyosin staining (blue); Col XII (green). (B, D, and F) AFOG staining of the corresponding sections used for immunostaining. (C) Col XII and Col Iα (red) are co-distributed in the post-cryoinjured area. (E) Col XII and Tenascin C (red) partially co-localize. (G) Fibronectin (red) partially overlaps with Col XII. N ≥ 4.

To genetically identify cardiac fibroblasts, we analyzed the cryoinjured hearts of transgenenic fish *wt1a(-6*.*8kb)*:*GFP*. We found that the transgene-expressing cells, which were present at the junction between compact and trabecular myocardium and in the cryoinjured zone, colocalized with Col XII fibrils (Fig 5A). We concluded that Col XII is produced by distinct cell populations, such as the epicardium, epicardial-derived cells and cardiac fibroblasts, indicating a heterogenous origin of collagenous matrix in the regenerating zebrafish heart.

### Col XII colocalizes with Col Iα in the post-cryolesion area

Mammalian and avian studies suggested that one function of Col XII is to regulate organization and mechanical properties of collagen fibril bundles to optimize the mechanical property of the connective tissues in conditions of high pressure [32, 42, 43]. Accordingly, we performed double immunofluorescence analysis between Col XII and other known matrix components of the fibrotic tissue, which is detected by histological staining in the post-cryolesion area (Fig 5B, 5D and 5F and S4 Fig). Unlike in the uninjured hearts, we found that Col XII and Col Iα displayed a nearly overlapping distribution in cryoinjured hearts, suggesting that they are part of the same network (Fig 5C). Interestingly, Col XII also partially co-localized with the tissue remodeling protein Tenascin C and with the adhesive matrix protein Fibronectin (Fig 5E and 5G). Thus, the fibrous tissue that repairs the damaged myocardial wall consists of a combination of various collagenous and non-collagenous matrix components, which might interact to ensure appropriate biomechanical properties of the beating zebrafish heart.

### Regulation of Collagen XII expression by TGF-β signaling in the post-cryolesion zone

**Fig 6:**
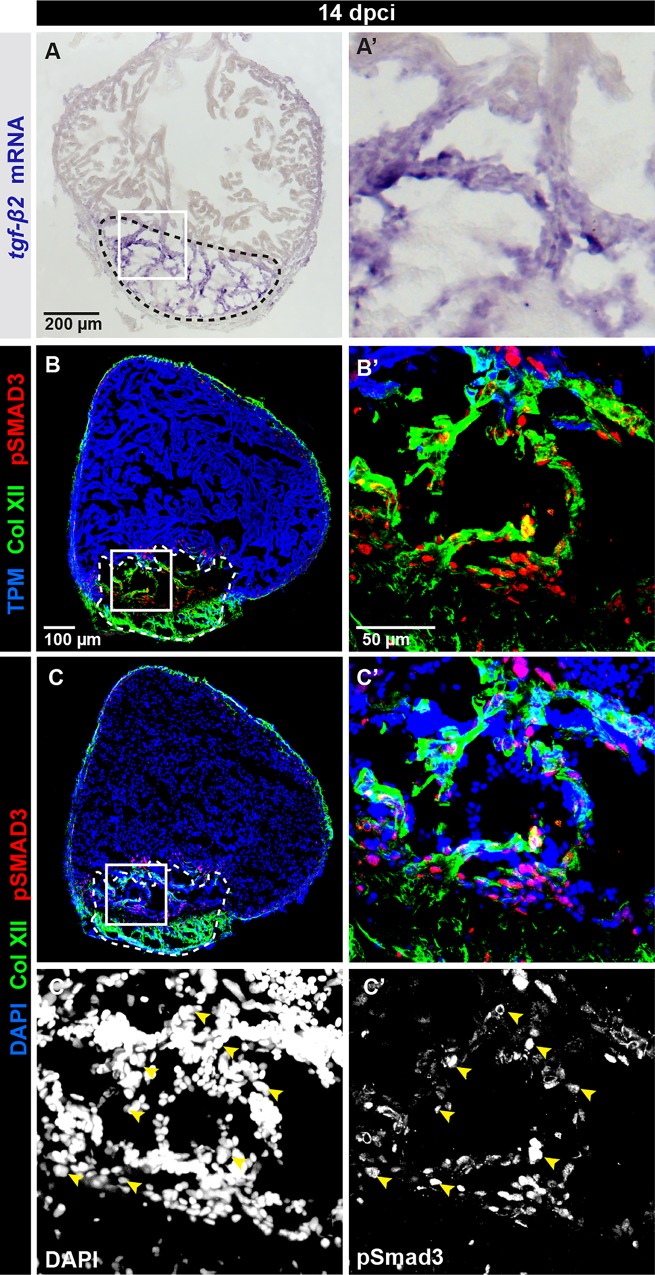
TGF-β signaling is activated in Collagen XII-positive fibrotic tissue. (A-C) Analyses of ventricle sections at 14 dpci. (A) *In -situ* hybridization detects *tgf-β2* expression in the cryoinjury area. N = 5. (B, C) Immunofluorescence staining reveals co-distribution of Col XII fibrils (green) and p-Smad3 (red) in the post-cryolesion zone that is recognized by the absence of Tropomyosin (blue in B). p-Smad3 reactivity is detected in the nuclei visualized by DAPI (blue in C). N = 5.

Next, we asked which regulatory mechanisms account for such an intense and specific expression of Col XII in the cryoinjured part. Our laboratory has previously characterized the role of TGF-β signaling in the stimulation of collagenous matrix deposition in the post-cryolesion zone [10]. *tgf-β* genes are expressed in the post-cryoinjured tissue, as exemplified by *tgf-β* at 14 dpci (Fig 6A). To investigate whether the Col XII expression pattern correlates with the TGF-β activity, we analyzed the presence of nuclear p-Smad3, which is an activated signal transducer of this pathway [44]. We found that p-Smad3 immunoreactivity was present in the fibrotic tissue containing Col XII, suggesting a possible link between TGF-β signaling and Col XII deposition (Fig 6B–6C’).

**Fig 7:**
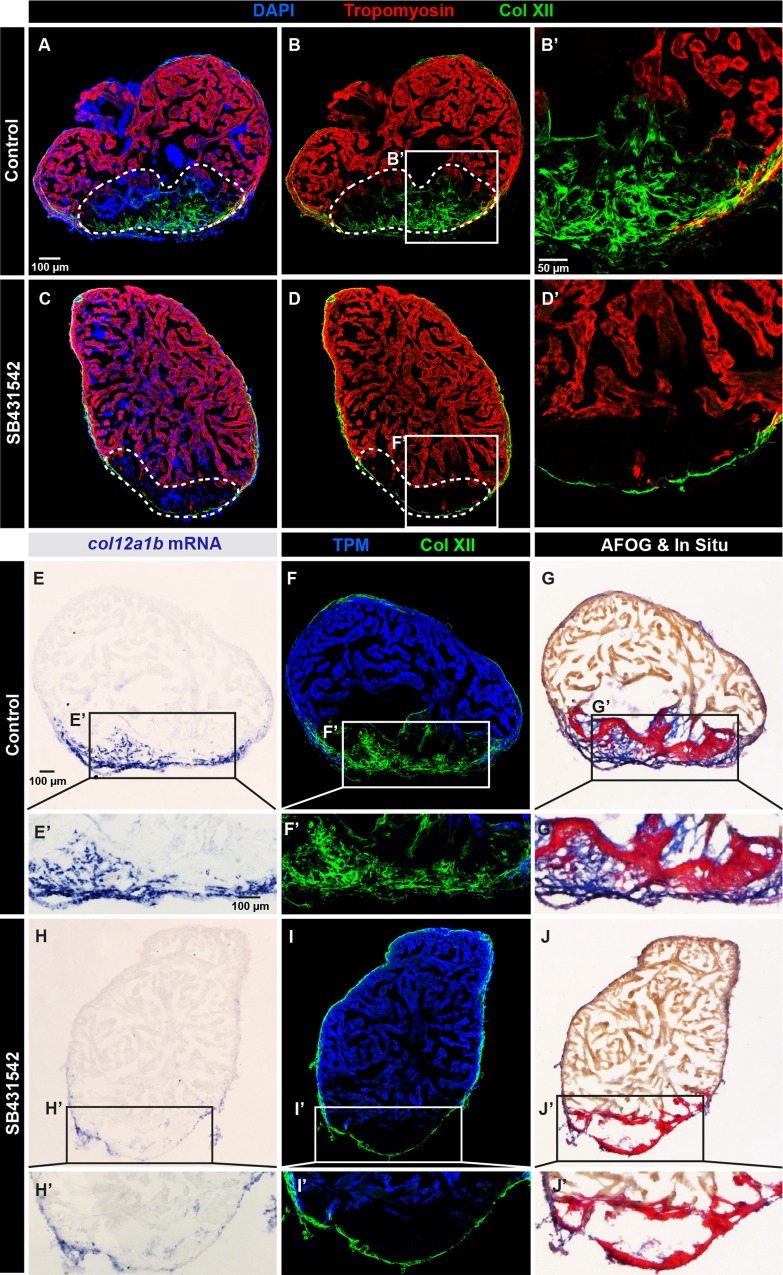
Collagen XII deposition in the post-cryolesion tissue requires TGF-β signaling. (A, B) Immunofluorescence staining shows a normal pattern of Col XII (green) in the Tropomyosin (red)-negative area in control heart. N = 5. (C, D) The treatment with the inhibitor of the TGF-β receptors, SB431542, suppresses the Col XII deposition (green) in the post-cryolesion area, without affecting the pre-existing epicardial fibrils. N = 7. (E-J) *In -situ* hybridization, double immunostaining and AFOG coloration of control (E-G) and SB431542-treated (H-J) hearts. (E, F) *In -situ* hybridization and fluorescent staining reveals that *col12a1b* transcripts (blue) match the distribution of Col XII protein. (F) Col XII fibrils (green) are present in the post-cryolesion area that is recognized by the absence of Tropomyosin (blue). (G) AFOG staining highlights the intact muscle (orange), fibrin-like matrix (red) and collagen (blue) that overlaps with the *in -situ* staining. (H) The inhibition of TGF-β signaling prevents upregulation of *col12a1b* expression in the post-cryoinjury area, without affecting its epicardial expression. (I) The cryoinjured zone is devoid of Col XII. (J) AFOG coloration reveals the absence of collagenous matrix (blue) in the post-infarcted zone. N = 6.

To perform a functional study, we used a specific pharmacological inhibitor of the TGF-β type I receptors, SB431542, which has been previously validated in the context of fin and heart regeneration in zebrafish [10, 24]. Treatment with 20 μM SB431542 was sufficient to significantly reduce phosphorylation of Smad3 in the heart, as compared to control hearts treated with 0.1% DMSO, which is the final concentration of the SB431542 solvent (S5 Fig). Remarkably, the exposure to 20 μM SB431542 suppressed Col XII expression in the post-cryoinjury zone without affecting the epicardial expression (Fig 7A–7D’).

Considering that matrix proteins are typically stable structures, they do not always reflect the actual transcriptional regulation. To examine the *col12a1b* gene expression, we performed *in-situ* hybridization that was followed by the sequential immunofluorescence assay and AFOG staining (Fig 7E–7J). In control hearts, *col12a1b* transcripts were detected in the cryoinjured part of the myocardium and in the epicardium, which corresponded to the pattern of Col XII protein distribution (Fig 7E–7G). The inhibition of TGF-β signaling resulted in the suppression of *col12a1b* expression specifically in the cryoinjured part without affecting the epicardial region (Fig 7H–7J). This suggests that the inductive mechanisms for Col XII expression might be differential in the outer and the inner tissue of the injured ventricle. However, we cannot exclude the presence of a residual TGF-β activity in the epicardium, despite the inhibitor treatment.

**Fig 8:**
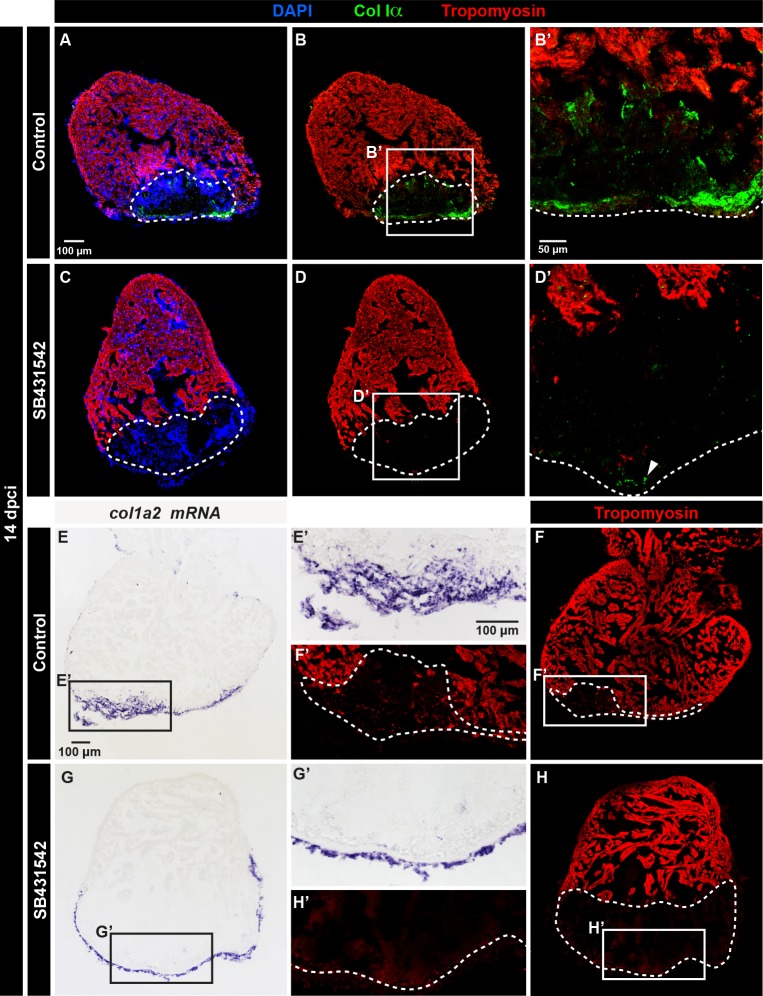
The upregulation of fibrillar Collagen Iα in the post-cryoinjured area is induced by TGF-β signaling. (A-D) Immunofluorescence staining of heart at 14 dpci using Col Iα (green) and Tropomyosin (red). The post-infarcted tissue is tropomyosin-negative (encircled with a dashed line). (A-B’) Control heart displays the presence of fibrillar collagen in the fibrotic tissue. N = 4.(C-D’) The treatment with the inhibitor of the TGF-β receptors, SB431542, suppresses Col Iα (green) in the inner wound site. In the epicardium, Col Iα can be still detected (arrowhead). N = 6. (E-H’) *In-situ* hybridization against *col1a2* (purple) and immunostaining with Tropomyosin (red). (E-F’) Control hearts display expression of *col1a2* in the post-infarcted tissue. (G-H’) The inhibition of TGF-β signaling with SB431542-treated suppresses *col1a2* expression in the inner part of the wound, without affecting the epicardial expression. N = 6.

Interestingly, we observed a similar effect on the deposition of fibrillar Col Iα, which was absent after the inhibition of the TGF-β signaling pathway (Fig 8A–8D). Consistently, *in-situ* hybridization analysis revealed a lack of *col1α2* transcription within the fibrotic tissue, although a normal expression was observed at the surrounding epicardium (Fig 8E–8H). Taken together, TGF-β signaling may directly or indirectly trigger both fibrillar Col Iα and non-fibrillar Col XII in the post-cryoinjured tissue, which repairs the damaged myocardial wall during heart regeneration.

## Discussion

Adult mammalian hearts fail to regenerate and heal by irreversible scarring, which is accompanied by cardiac hypertrophy and dilation. By contrast, zebrafish cardiomyocytes remain competent for dedifferentiation and proliferation during the entire lifespan, while the reparative fibrotic tissue beneficially supports the damaged ventricle, being able to progressively resolve in order to give space for new cardiac muscle. Which collagenous components are unique to this reversible fibrotic and dynamic epicardial matrix that render it so suitable for regenerative processes? In this study, we identified that FACIT-type Col XII, is expressed in the intact epicardial region and becomes abundantly upregulated in the reparative tissue. FACIT collagens play a particular role in the matrix, because they do not form fibers on their own, but they regulate fibril size and spacing, as well as link collagenous fibrils to each other, and to other ECM components [14, 45]. Col XII is thought to modulate the matrix arrangement and its morphogenetic flexibility, especially under biophysical stress [16]. This function is particularly important during organogenesis, and consistently, Col XII deposition is abundant in embryonic tissues of fish and tetrapods [16, 20]. To our knowledge there are no reports of Col XII deposition in either the mammalian epicardium or a fibrotic scar. Thus, the presence of this FACIT protein in the zebrafish analogous tissues might significantly influence the matrix properties that are particularly beneficial for heart regeneration.

Despite a common origin of the vertebrate heart, substantial structural differences are apparent between the adult myocardium of fish and mammals. One of the main anatomical distinctions is the architecture of the heart chambers. In contrast to the human heart, the zebrafish heart is composed mainly of a trabecular myocardium that is surrounded by a thin compact layer [46]. A recent electron microscopy study provided evidence that both myocardial compartments are interconnected by a complex junctional region, which contains a network of flattened fibroblasts and collagen fibrils [29]. Here, we show that this region is demarcated by *wt1a(-6*.*8kb)*:*GFP* positive fibroblasts and Col XII-positive fibrils. Thus, Col XII might contribute to the flexible matrix that links differently oriented cardiac muscle cells of the compact and trabecular myocardium. Further studies are required to determine the role of Col XII in the junctional regions of the zebrafish heart.

**Fig 9:**
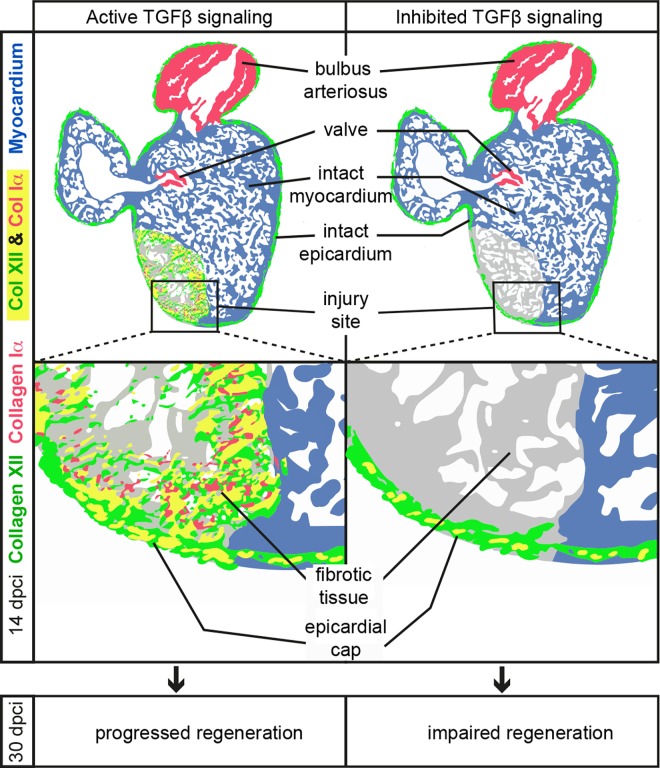
Schematic representation of distinct effects of TGF-β inhibition on Col XII and Col Iα deposition in the epicardium and the fibrotic tissue during zebrafish heart regeneration. Illustrations of longitudinal heart sections at 14 dpci in normal conditions (left side) and after inhibition of the TGF-β signaling pathway (right side). The uninjured part of the heart displays the presence of Col XII along the heart surface, while Col Iα is expressed in the bulbus arteriosus and in the atrio-ventricular valves. The injured myocardial wall heals by enhanced Col XII deposition along the outer margin of the wound, forming a Col XII-rich epicardial cap. The inner part of the damaged myocardium is replaced with fibrotic tissue that contains *tgf-β*-expressing cells. The activity of this pathway stimulates deposition of fibrillar Col Iα and fibril-associated Col XII in the fibrotic tissue, but it is not required for the formation of the epicardial cap. The provisional matrix maintains the organ function during the regenerative process, until its completion at 30 dpci.

The main focus of this study was on the accumulation of Col XII during heart regeneration. Our immunofluorescence analysis revealed that Col XII tightly colocalized with fibrillar Col Iα and several other glycoproteins at the wound site of the ventricular wall. To obtain mechanistic insights into the regulation of Col XII expression, we investigated the impact of TGF-β signaling on the induction of *col12* and *col1a* after myocardial infarction. We found that the TGF-β signaling pathway is required for the upregulation of both these genes in the inner injury zone of the ventricle. By contrast, the epicardial expression of *col12* and *col1a* were not affected by the TGF-β inhibition (Fig 9). Thus, the regulation of collagens is differential in mesothelial and fibrotic tissues of the regenerating zebrafish heart.

A multi-factorial regulation of Col XII expression has also been shown in mammalian systems. In tenocyte cultures, TGF-β signaling has been shown to upregulate *col12* expression [47, 48]. There are also several lines of evidence from *in-vivo* and *in-vitro* studies that tensile force stimulates Col XII deposition [16]. It is likely that the mechanisms controlling the expression level of the FACIT protein involve a combination of tissue-specific transcriptional regulators, paracrine signals and biomechanical cellular sensors. Further biochemical and transgenic studies of the *col12* promoter will be required to dissect the variety of regulatory responsive elements. The elucidation of the mechanisms regulating the expression of FACIT proteins in specific tissues is essential to understand matrix biology during organ development and regeneration.

### Association between fibrillar Col Iα and non-fibrillar Col XII

Our *in-situ* hybridization analysis revealed that two homologous FACIT collagens, *col12a1a* and *col12a1b*, and the fibril-forming collagens, *col1a1a* and *col1a2*, are co-expressed in the tissue sheath covering the intact zebrafish ventricle. Despite a partially overlapping transcription pattern of both genes on the heart surface, antibody staining against Col Iα and Col XII failed to reveal a co-localization of both proteins. Furthermore, the immunoreactivity appeared to be nearly exclusive: Col XII labeling was restricted to the superficial layers of all heart structures, while Col Iα staining filled the interstitial matrix of non-muscular structures. A differential Col Iα and Col XII protein localization was also reported during atrio-ventricular valve remodeling in mice [43]. The discrepancy between *in-situ* hybridization and immunofluorescence staining is not unusual for matrix components, and can be attributed to differences in transcript stability and protein turnover, as well as to posttranslational modifications, multimerization and agglomeration with other partner molecules, which might interfere with the exposition of immunoreactive epitopes. In amniotic vertebrates, immunogold and immunofluorescence labeling provided evidence that Col XII tightly associates with collagenous fibril surfaces and with basement membranes [20, 31, 32, 49, 50]. Thus, it is possible that Col XII proteins that are bound to the surface of Col Iα-fibrils could hinder the accessibility of certain Col Iα epitopes for immunoreactivity. Further studies are required to investigate interactions between zebrafish fibril-forming and fibril-associated collagens.

### Col XII contribution to the provisional scaffold of the regenerating heart

Col XII deposition is dynamically regulated in the post-infarcted tissue. Importantly, the cryoinjury procedure did not destroy the original collagenous layer of the epicardium. This is an expected finding, as membrane-independent structures are more resistant to freezing/thawing than cells. After death of cardiac cells, the persisting ECM could serve as a ready fibrous skeleton for the establishment of transient connective tissue during the reparative phase. Indeed, previous characterization of the post-cryoinjury tissue revealed a trabecular organization of the fibrotic scar tissue that emerges after resorption of fibrin and dead cells [6–9]. Here, we identified that Col XII accumulates in the post-injured area with the most abundant appearance at 14 dpci. Importantly, Col XII fibers closely associated with cardiomyocytes of the regenerative leading edge. Thus, Col XII could be involved in bridging the provisional fibrotic tissue to the regenerating edge of the myocardium. This could be achieved indirectly, through binding to other adhesion and de-adhesion matrix proteins, such as Fibronectin and Tenascin C, both of which partially co-localize with Col XII. This finding is consistent with studies in other species, in which Col XII was found to bind collagenous fibrils and other matrix components, namely Decorin, Tenascin X, Fibromodulin and Cartilage oligomeric matrix protein COMP [32, 50–53]. Thus, in the zebrafish heart, as in mammalian tissues, Col XII might interact with various matrix components to generate unique biomechanical properties in the post-cryolesion tissue. Further functional studies are required to address the function of this FACIT protein during heart regeneration.

## Supporting Information

S1 Fig The adult zebrafish heart and its collagenous components. (TIF)

S2 Fig Croyinjury does not damage the pre-exisiting Col XII protein in the epicardium. (TIF)

S3 Fig *col12a1a*-expressing cells accumulate at the site of injury at the onset of heart regeneration. (TIF)

S4 Fig Complete ventricle sections that were used for analysis of fibrotic tissue at 14 dpci in Fig 5. (TIF)

S5 Fig Treatment with 20 μM SB431542 reduces the activity of the TGF-β signaling pathway in injured hearts. (TIF)
