# Supplementary figures and images for: Collagen XII Contributes to Epicardial and Connective Tissues in the Zebrafish Heart during Ontogenesis and Regeneration
Source: PLoS One. 2016 Oct 26;11(10):e0165497. doi: 10.1371/journal.pone.0165497 (PMC5081208; doi:10.1371/journal.pone.0165497)

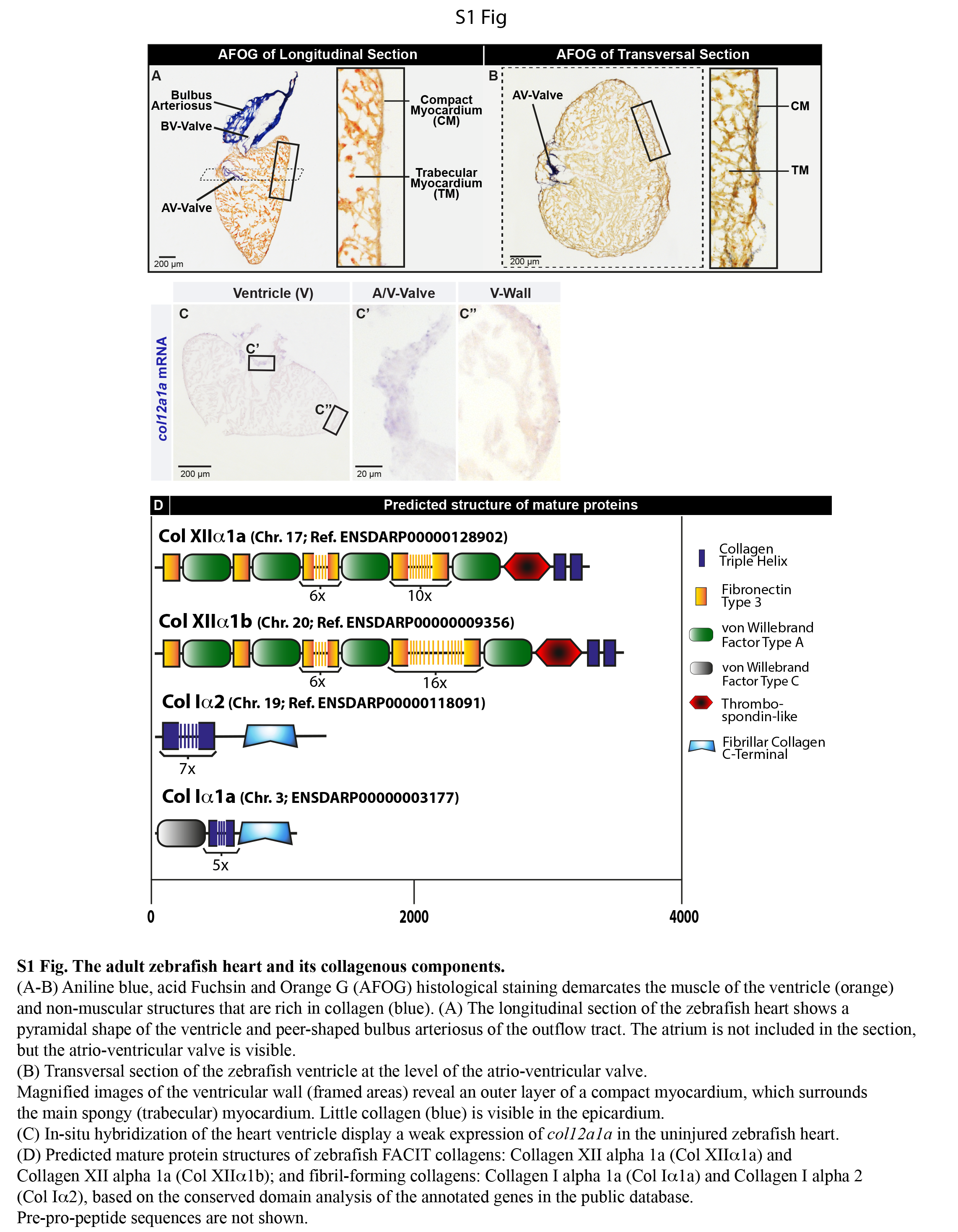

Supplement: S1 Fig — (TIF) [file pone.0165497.s001.tif]

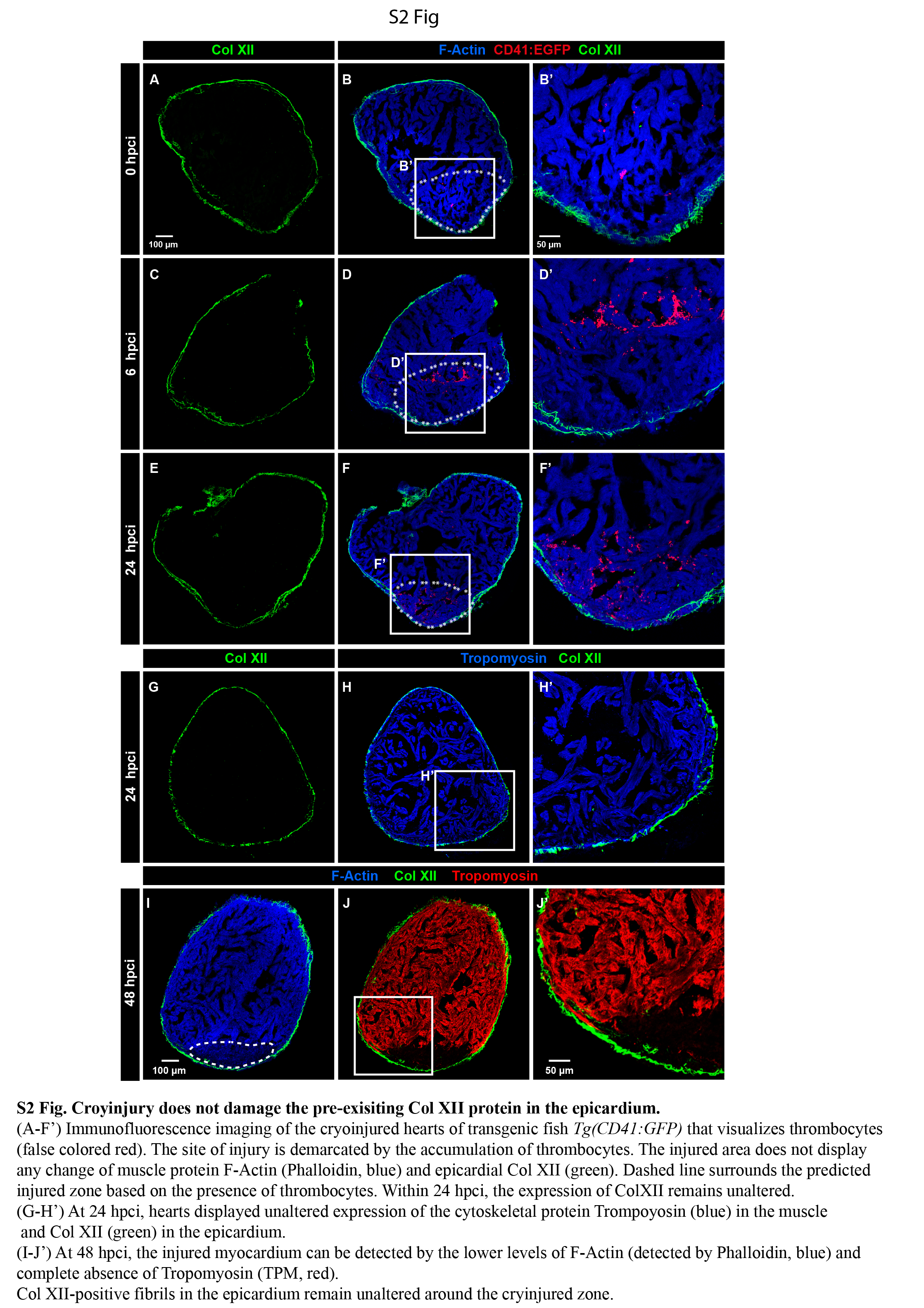

Supplement: S2 Fig — (TIF) [file pone.0165497.s002.tif]

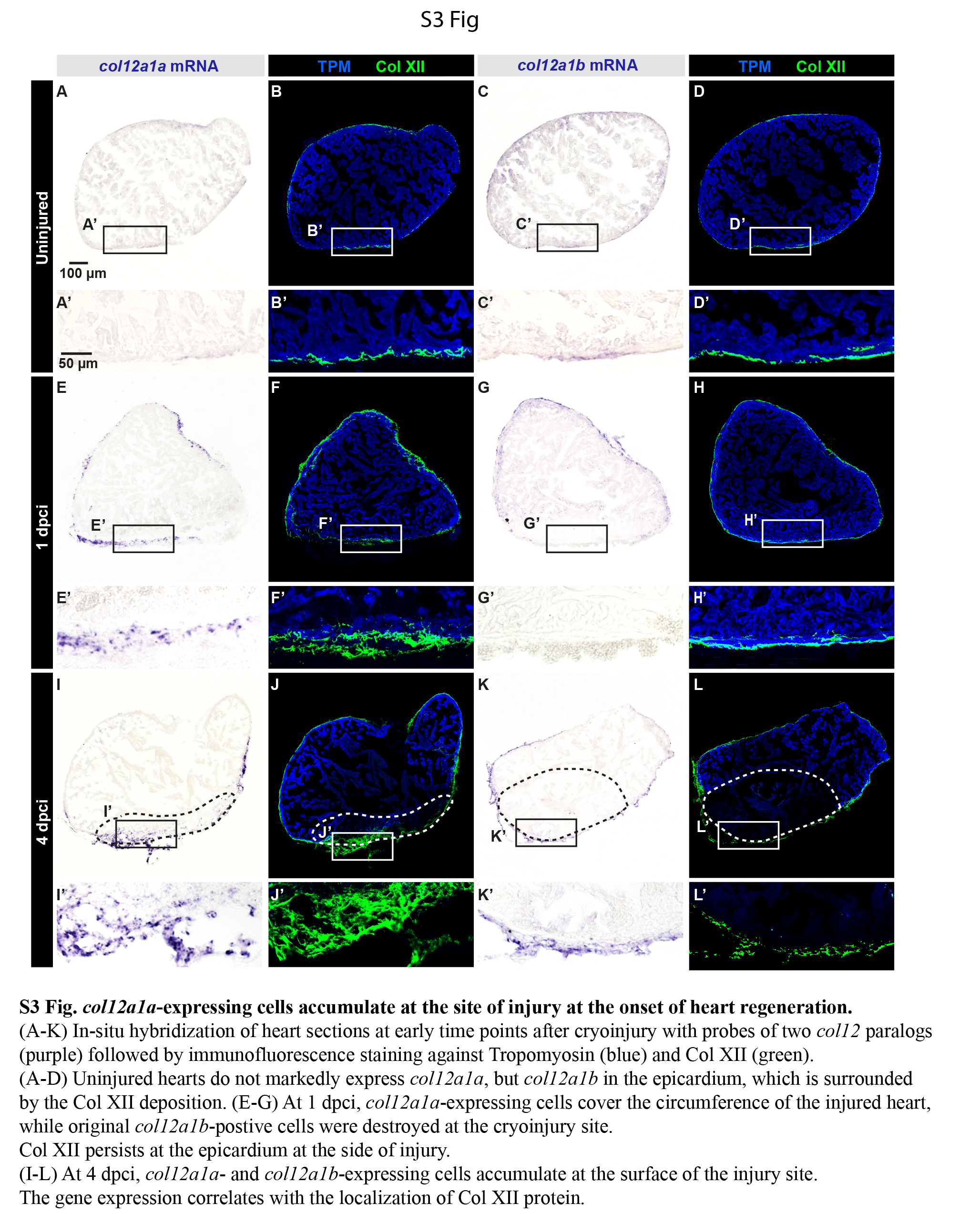

Supplement: S3 Fig — (TIF) [file pone.0165497.s003.tif]

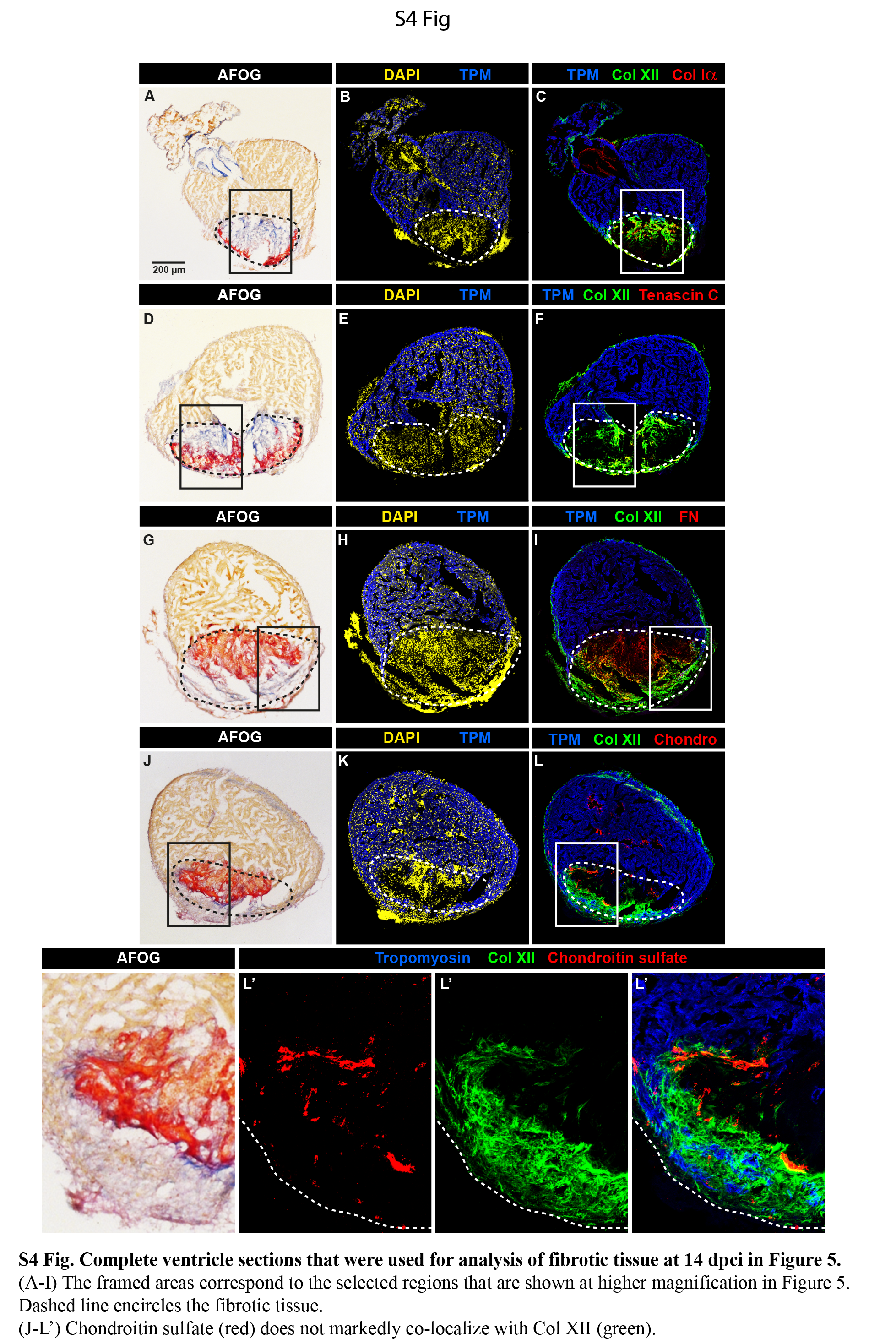

Supplement: S4 Fig — (TIF) [file pone.0165497.s004.tif]

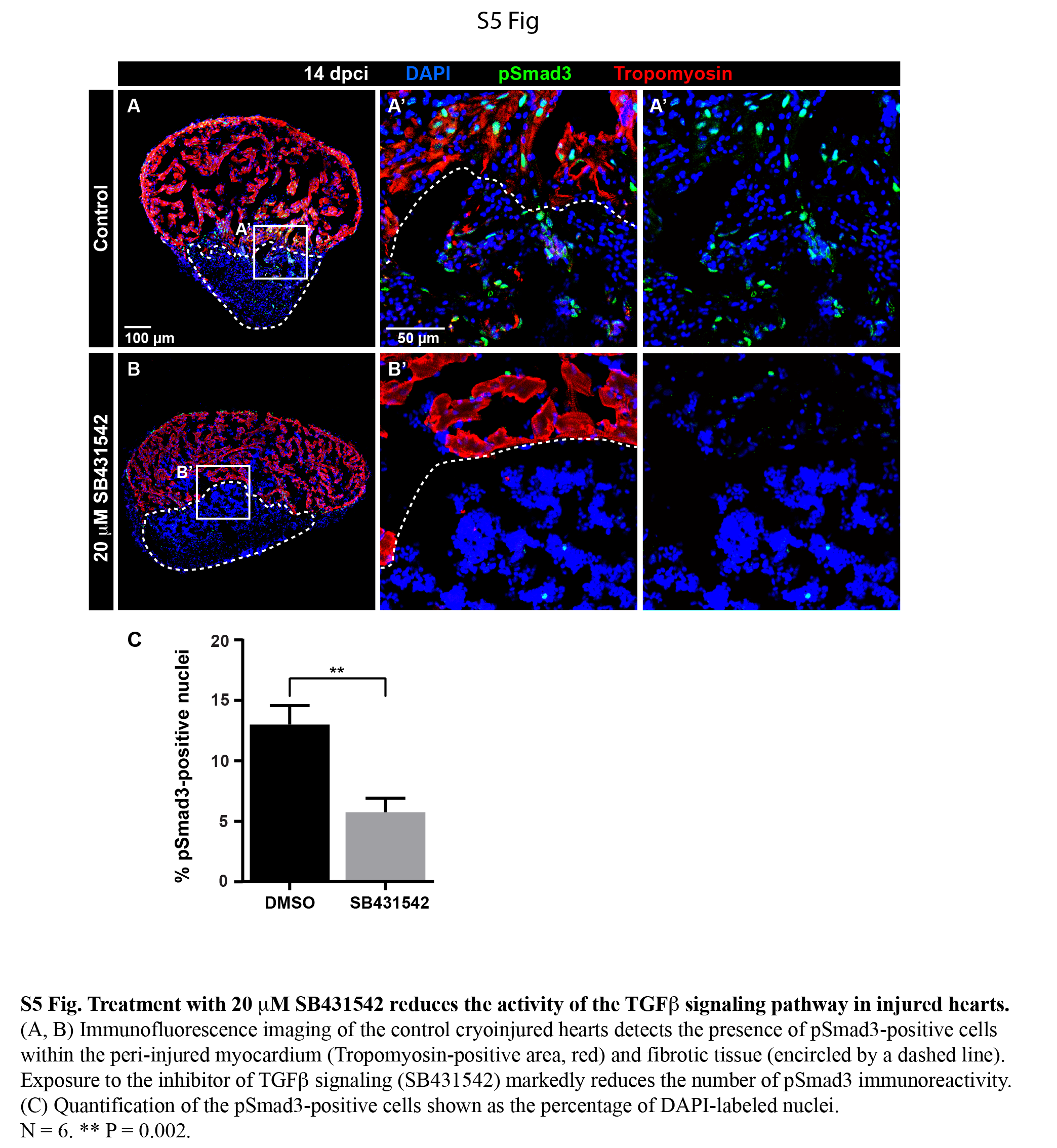

Supplement: S5 Fig — (TIF) [file pone.0165497.s005.tif]
